# Silicon dioxide nanoparticles induced neurobehavioral impairments by disrupting microbiota–gut–brain axis

**DOI:** 10.1186/s12951-021-00916-2

**Published:** 2021-06-10

**Authors:** Jun Diao, Yinyin Xia, Xuejun Jiang, Jingfu Qiu, Shuqun Cheng, Junhao Su, Xinhao Duan, Min Gao, Xia Qin, Jun Zhang, Jingchuan Fan, Zhen Zou, Chengzhi Chen

**Affiliations:** 1 Department of Occupational and Environmental Health, School of Public Health and Management, Chongqing Medical University, Chongqing, 400016 People’s Republic of China; 2 Center of Experimental Teaching for Public Health, Experimental Teaching and Management Center, Chongqing Medical University, Chongqing, 400016 People’s Republic of China; 3 Department of Health Laboratory Technology, School of Public Health and Management, Chongqing Medical University, Chongqing, 400016 People’s Republic of China; 4 Department of Pharmacy, The First Affiliated Hospital of Chongqing Medical University, Chongqing, 400016 People’s Republic of China; 5 Molecular Biology Laboratory of Respiratory Disease, Institute of Life Sciences, Chongqing Medical University, Chongqing, 400016 People’s Republic of China; 6 Dongsheng Lung-Brain Disease Joint Lab, Chongqing Medical University, No. 1, Yixueyuan Road, Yuzhong District, Chongqing, 400016 People’s Republic of China

**Keywords:** Silicon dioxide nanoparticles, Gut–brain axis, Gut microbiota, Neurobehavioral impairments

## Abstract

**Background:**

Silicon dioxide nanoparticles (SiO_2_NPs) are widely used as additive in the food industry with controversial health risk. Gut microbiota is a new and hot topic in the field of nanotoxicity. It also contributes a novel and insightful view to understand the potential health risk of food-grade SiO_2_NPs in children, who are susceptible to the toxic effects of nanoparticles.

**Methods:**

In current study, the young mice were orally administrated with vehicle or SiO_2_NPs solution for 28 days. The effects of SiO_2_NPs on the gut microbiota were detected by 16S ribosomal RNA (rRNA) gene sequencing, and the neurobehavioral functions were evaluated by open field test and Morris water maze. The level of inflammation, tissue integrity of gut and the classical indicators involved in gut–brain, gut–liver and gut–lung axis were all assessed.

**Results:**

Our results demonstrated that SiO_2_NPs significantly caused the spatial learning and memory impairments and locomotor inhibition. Although SiO_2_NPs did not trigger evident intestinal or neuronal inflammation, they remarkably damaged the tissue integrity. The microbial diversity within the gut was unexpectedly enhanced in SiO_2_NPs-treated mice, mainly manifested by the increased abundances of *Firmicutes* and *Patescibacteria*. Intriguingly, we demonstrated for the first time that the neurobehavioral impairments and brain damages induced by SiO_2_NPs might be distinctively associated with the disruption of gut–brain axis by specific chemical substances originated from gut, such as *Vipr1* and *Sstr2*. Unapparent changes in liver or lung tissues further suggested the absence of gut–liver axis or gut–lung axis regulation upon oral SiO_2_NPs exposure.

**Conclusion:**

This study provides a novel idea that the SiO_2_NPs induced neurotoxic effects may occur through distinctive gut–brain axis, showing no significant impact on either gut–lung axis or gut–liver axis. These findings raise the exciting prospect that maintenance and coordination of gastrointestinal functions may be critical for protection against the neurotoxicity of infant foodborne SiO_2_NPs.

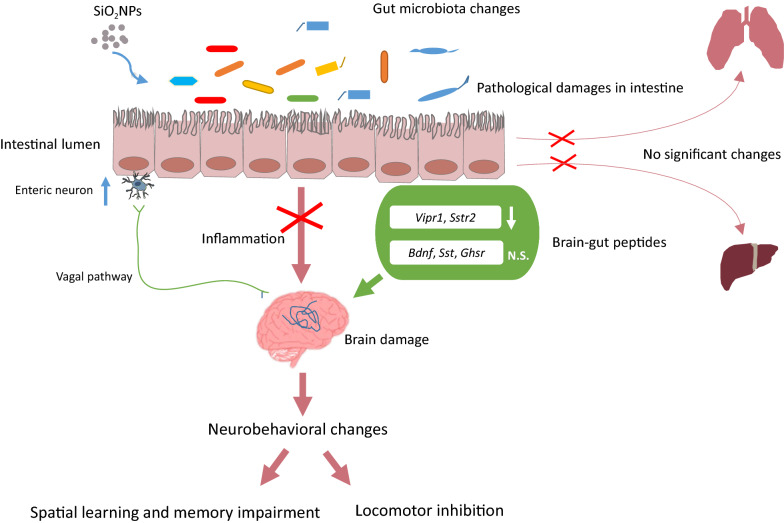

**Supplementary Information:**

The online version contains supplementary material available at 10.1186/s12951-021-00916-2.

## Introduction

Silicon dioxide (SiO_2_) is a natural chemical that most widely used in structural materials, microelectronics, and as components in the food industry for various applications [1–4]. The amorphous form of SiO_2_, also known as synthetic amorphous silica (SAS), is authorized as a food additive coded E551 [3, 5]. It usually functions as an anti-caking agent to maintain free-flowing properties of powdery products, preserve food color during storage, and carry fragrances or flavors. It is noteworthy that SiO_2_ can exist in various particle sizes in the food additive depending on the manufacturing process [6]. For instance, the sizes of SiO_2_ in the E551 contains primary particles in the nano-size range, and most of the particles are normally greater than 100 nm. However, under the conditions present in the gastrointestinal tract, SiO_2_ particles are capable of clumping together and subsequently degrading into small size particles, many of which are nanoparticles with size less than 100 nm, approximately 10–50 nm in size [3, 7, 8]. The nano-size SiO_2_ in the E551 may have a completely different influence on the uptake and distribution of SiO_2_ within the body [4]. Although SiO_2_ is used as a classical food additive for a long history without any detrimental health effects, the safety concerns should be still paid on the potential health risks for humans associated with nanoparticles in E551.

The potential toxic effects of nano-size SiO_2_ have been extensively studied for years [3, 4, 9, 10]. Despite these nanoparticles are generally considered less harmful in the past decade, excessive exposure to SiO_2_ nanoparticles (SiO_2_NPs) has been recently reported to cause injuries to cells, tissues, and organs in vitro and in vivo [10–12]. Most of in vitro studies demonstrate that SiO_2_NPs are able to induce size-, shape- and dose-dependent cytotoxic effects in cultured human cell lines, such as glioblastoma cells [13], A549 cells [14] and BEAS-2B cells [15] etc. Evidence from scarce in vivo investigations illustrate that consumption of very large quantities of SiO_2_NPs can result in adverse effects in liver, kidney, and lung of animals [11, 16, 17]. Notably, if SiO_2_NPs are injected directly into the bloodstream, even small quantities are harmful for the experimental animals [3, 11, 18, 19]. Inhalation exposure to SiO_2_NPs is highly associated with human health, especially occurs in occupational environment [20]. Moreover, either inhaled or ingested SiO_2_NPs can penetrate cells and interact with cellular membrane or organelles to trigger mammalian cell death by induction of oxidative stress, endoplasmic reticulum stress and apoptosis [21–23].

Studies have demonstrated that exposure to SiO_2_NPs leads to the observable effects on the alterations of behavioral phenotypes in zebrafish, such as disturbance on light/dark preference, abnormal exploratory behavior and deficits in memory [24]. Even at low dose level, SiO_2_NPs have also been shown to increase the apoptotic cells in the central nervous system of zebrafish embryos and disrupt the axonal integrity [25]. Similarly, after treating of SiO_2_NPs by intranasal instillation for more than 30 days, exposed mice exhibited obvious mood dysfunction, cognitive impairment and neurodegeneration-like pathology [26]. In cultured neuroblastoma SH-SY5Y cells, treatment of SiO_2_NPs causes deleterious effects on tau structure and cell integrity [27]. These results are verified in other type of neuronal cells, showing that exposure to SiO_2_NPs induces pathological signs of Alzheimer’s disease, such as changed expression of amyloid precursor protein, increased phosphorylation of tau in neuro2a neuroblastoma cells [28]. The evidence obtained from cultured cells and animals together suggest that exposure to SiO_2_NPs can trigger neurotoxic effects and may be considered as a risk factor facilitating the neurological disorders onset and/or progression. Therefore, currently, increasing concerns have been raised over the potential neurotoxicity of SiO_2_NPs on human health due to their extensively use as food additive. However, whether oral exposure to SiO_2_NPs may induce neurotoxic effects and their underlying mechanisms remain largely unknown.

Oral uptake is considered as the major route of exposure to SiO_2_NPs for general population [3, 6, 11]. Following ingestion, SiO_2_NPs interact with the complex gastrointestinal microenvironment. They are able to accumulate in the gastrointestinal tract as a result of daily consumption and affect the gut microbiota and mucus layer directly [6]. A portion of SiO_2_NPs possibly show a significant impact on the enteric neurons when translocating through the epithelial barrier, before reaching systemic circulation [6]. It has been noted that the gut microbiota plays a critical role in the regulation of brain functions as indispensable substrate for host health. The imbalance of intestinal microbial ecosystem may strongly contribute to the neurobehavioral impairments via bidirectional gut–brain communication [29]. Many neurological diseases are closely related with changes along the microbiota–gut–brain axis, such as Alzheimer’s disease [30] and Parkinson’s disease [31]. However, whether oral exposure to SiO_2_NPs facilitates the onset of neurological disorders via microbiota–gut–brain axis has not been reported yet.

Therefore, in this study, we aimed to verify if oral exposure of SiO_2_NPs induced neurobehavioral impairments through disruption of microbiota–gut–brain axis. Since children were usually susceptible to the neurotoxic effects of exogenous chemicals, young animals were subjected to intragastric administration of SiO_2_NPs for 28 days. Herein, our data demonstrated for the first time that, the neurobehavioral impairments induced by SiO_2_NPs treatment possibly occurred by distinctively damaged the microbiota–gut–brain axis but showed no significant impact on either gut–liver or gut–lung axis. These findings will provide us a novel insight that dietary exposure to SiO_2_NPs may potentially disturb the intricate dialogue between gut and brain functions, hence resulting in the neurotoxicity. The health risk of foodborne SiO_2_NPs should be reconsidered, especially for the infants and children, who are more sensitive to the neurotoxic effects of SiO_2_NPs than adults.

## Materials and methods

### Chemical and reagents

SiO_2_NPs nano-powder were obtained from Sigma Aldrich Chemical Co. (MO, USA, Cat Number: 637246). 4ʹ,6-Diamidino-2-phenylindole (DAPI) was from Beyotime Institute of Biotechnology (Shanghai, China). Hematoxylin–eosin, Toluidine blue O (TBO) and Alcian blue periodic acid schiff (AB-PAS) staining kits were all purchased from Solarbio Science & Technology Co., Ltd. (Beijing, China). Antibodies against Hu protein C/D (HuC/D), neuron-specific class III beta-tubulin (TuJ1) and anti-lysozyme were purchased from Abcam Co., (Cambridge, UK). Mouse secretory immunoglobulin A (sIgA) and mouse diamine oxidase (DAO) enzyme-linked immunosorbent assay (ELISA) kits were from Cusabio Biotech Co. Ltd. (Wuhan, China). The commercial kits of determining sucrase, lactase, maltase, alkaline phosphatase, γ-glutamyl transferase, superoxide dismutase (SOD) activities and malondialdehyde (MDA) contents were obtained from Nanjing Jiancheng Bioengineering Institute Co., Ltd. (Nanjing, China).

### Animals

A total of 20 healthy male specific pathogen-free C57BL/6J mice, aged 4 weeks and weighted 8–12 g, were provided by Experimental Animal Center of Chongqing Medical University [Chongqing, China, License numbers: SCXK(Yu)2018-0003]. Animals were housed under constant conditions with room temperature at 23 ± 1 °C and humidity at 55 ± 10%. They were all maintained in a standard 12 h:12 h light–dark cycle. The mice were free access to laboratory mouse chow and tap water. The animals were randomly assigned into vehicle group and SiO_2_NPs-treated group according to the website https://www.randomizer.org. Each group had ten animals. The use of animals in experimental research were approved by the Institutional Animal Care and Use Committee of Chongqing Medical University, and all efforts were made to minimize the pain or distress experienced by animals. This study received ethical approval from The Ethical Committee of Chongqing Medical University.

### Characterization and preparation of SiO_2_NPs

**Fig. 1:**
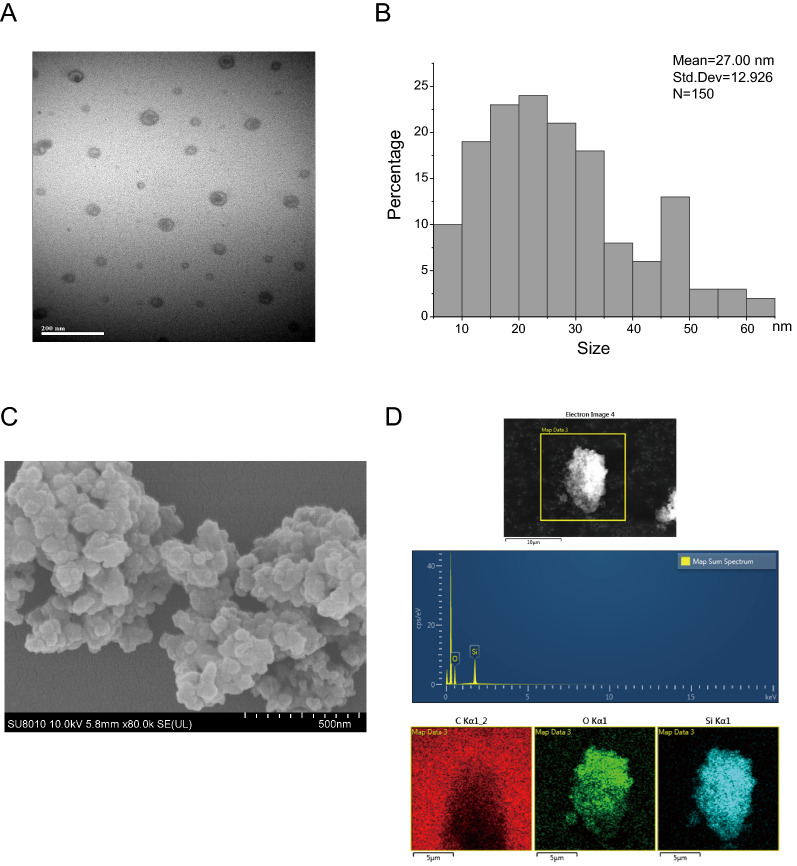
Characteristics of SiO_2_NPs used in this study. **A** Transmission electron microscope (TEM) image of SiO_2_NPs was shown. Scale bar, 200 nm. **B** The average size of SiO_2_NPs was around 27 ± 12.926 nm calculated from 150 nanoparticles in TEM images. **C** Scanning electron microscope image of SiO_2_NPs. Scale bar, 500 nm. **D** Chemical composition of SiO_2_ was detected by FE-SEM/EDS. Scale bar, 5 μm

The powder of SiO_2_NPs were diluted in sterile physiological saline solution and sonicated with an ultrasonic cleaner (SB-5200DT, Ningbo Scientz Biotechnology Co., Ltd, Ningbo, China) on ice at 20% of maximum amplitude for 20 min. The suspended solution of SiO_2_NPs was freshly prepared for each time use. The surface area was 590–690 m^2^/g and the purity was 99.5% according to the instruction from manufacture. The morphology of SiO_2_NPs was observed by a transmission electron microscope (TEM) (Hitachi-7500, Hitachi, Ltd, Tokyo, Japan). A total of 150 nanoparticles were counted and the average size of nanoparticle was calculated. The average particle size of SiO_2_NPs used in current study was (27 ± 12.926) nm. The field emission scanning electron microscopy (Hitachi-SU8010, Hitachi, Ltd, Tokyo, Japan) with energy-dispersive spectroscopy (Oxford X-MAN 50) (FE-SEM/EDS) was used to determine the chemical elemental composition of SiO_2_NPs. The characteristics of SiO_2_NPs were shown in Fig. 1A–D. According to the results of previous study, the hydrodynamic diameter of SiO_2_NPs used in this study was 471 ± 169 nm, the PDI was 0.48 and the zeta-potential was − 31.3 ± 1.8 mV [32]. The surface area for SiO_2_NPs was 500–840 m^2^/g detected by Brunauer–Emmett–Teller nitrogen adsorption [32, 33].

### Rationale for SiO_2_NPs’ dose selection and treatment

The dose of SiO_2_NPs used in this study were calculated according to the Chinese Standard for Food Additives (GB2760-2014). The calculation methods were described in detail as follows. It declared that the upper limit of SiO_2_ for using in food additives was 20 g/kg in the national standard of GB2760-2014. Herein, we assumed that the weight of each bag of infant food was 80 g and the weight of a 1-year-old infant was 10 kg. If each infant was fed with one bag of food each morning and evening twice a day. The total daily intake of SiO_2_ for each infant was 0.32 g/kg [(80 g × 2 ÷ 1000 g) × 20 g ÷ 10 kg = 0.32 g/kg]. Since the equivalent dose conversion from mouse to human was near 9.1-fold [34], the dosage of SiO_2_ was calculated from the following formula: 0.32 g/kg × 9.1 = 2.912 g/kg ≈ 3 g/kg. Thus, animals were treated with either vehicle solution or with 3 g/kg SiO_2_NPs suspension solution once a day via intragastric administration between 9:30 A.M. and 10:30 A.M. and lasted for 28 days. The number of cells in the bronchoalveolar lavage fluid were counted using TC20^TM^ Automated Cell Counter (Bio-Rad, Hercules, CA, USA).

### Hematoxylin–eosin staining

Hematoxylin–eosin (H&E) staining was carried out according to the protocols described previously [35]. In brief, after designed treatment, the animals were sacrificed by cervical dislocation under anesthesia. The intestine and brain tissues were quickly dissected and fixed in fresh prepared 4% paraformaldehyde. The sections were subsequently dewaxed in xylene and dehydrated by ethanol, followed by staining with hematoxylin and eosin. After mounting with neutral balsam, the sections were observed under an Olympus light microscope (IX53, Tokyo, Japan).

### Alcian blue periodic acid schiff (AB-PAS) staining

Goblet cells were stained by AB-PAS according to the procedures described previously [36]. Briefly, after treatment, the intestine tissue was collected and immersed into fresh prepared 4% paraformaldehyde. The sections were then dehydrated in the ethanol followed by staining with alcian blue solution, Schiff Reagent and hematoxylin, respectively. The sections were then subjected to the ethanol dehydration process and mounted with neutral balsam. Finally, the sections were observed under a light microscope (Olympus, IX53, Tokyo, Japan).

### Toluidine blue staining

Mast cells were stained by Toludine blue according to the procedures reported previously [37]. In brief, the intestine tissue was dissected and fixed in fresh prepared 4% paraformaldehyde. The sections were then deparaffinized by xylene, dehydrated in the ethanol followed by staining with toluidine blue solution. After mounting with the neutral balsam, the sections were observed under an Olympus light microscope (IX53, Tokyo, Japan).

### Immunofluorescence assay

Immunofluorescence assay was performed according to the protocols reported previously [38]. Briefly, the intestine tissue was collected immediately and fixed in fresh prepared 4% paraformaldehyde. Hereafter, the sections were washed with phosphate buffer solution and blocked in the normal serum for 30 min. After incubation with the primary antibodies against TuJ1 (1:250) and HuC/D (1:250) overnight at 4 °C, the sections were then incubated with fluorescent dye-conjugated secondary antibodies for 1 h. Finally, the sections were stained with DAPI, and observed under a fluorescence microscope (Olympus, IX53, Tokyo, Japan). The fluorescence intensity was measured by using Image-Pro Plus 6.0 software (Bethesda, MD, USA).

### Immunohistochemistry assay

Immunohistochemistry assay was carried out according to the procedures reported previously [39]. In brief, the intestine tissue was collected and immersed into fresh prepared 4% paraformaldehyde. The sections were deparaffinized in xylene and subjected to conventional gradient ethanol dehydration. Subsequently, the fresh 3% hydrogen peroxide was used to inhibit the activity of endogenous tissue peroxidase. After washing thoroughly with phosphate buffer solution, the sections were incubated in the block solution containing serum for 30 min followed by incubation of anti-lysozyme primary antibody (1:1000) at 4 °C overnight. Next day, the sections were incubated with biotinylated secondary antibody and horseradish enzyme-labeled streptavidin for 10 min, respectively. The positive reactions in the tissues were visualized by a freshly prepared diaminobenzidine. The sections were then observed under a light microscope (Olympus, IX53, Tokyo, Japan).

### Quantitative PCR assay

Quantitative PCR assay was conducted according to the protocols reported previously [40]. Briefly, the tissues of cortex, lung, intestine, liver were quickly collected and stored at − 80 °C. Total RNA was isolated by using TRizol method. Complementary DNA (cDNA) was synthesized using Perfect Real Time PrimeScript™ RT Master Mix. Quantitative RT-PCR was performed with TBGREEN Premix Ex Taq™ II (TliRNaseH Plus) on the CFX Connect™ Real-Time System (Bio-Rad, Hercules, CA, USA). The specific primers were synthesized by Sangon Biotech, Co., Ltd. (Shanghai, China), and the sequences of target genes were listed in Additional file 2: Table S1. The amplifications were carried out according the conditions shown as follows, 95 °C for 2 min, followed by amplification in 40 cycles of 95 °C for 5 s, 15 s at 60 °C and 20 s at 72 °C, then 65 °C and 95 °C for 5 s. The relative mRNA expressions of target genes were normalized by the relative amount of β-actin mRNA.

### Measurement of sucrase, lactase, maltase, alkaline phosphatase, γ-glutamyl transferase, SOD activities and MDA contents

The tissues of intestine, lung and liver were quickly collected from each mouse at the end of treatment and stored at − 80 °C before determination. The tissues were then homogenized in 0.9% sodium chloride solution using an electric glass homogenizer. The activities of sucrase, lactase, maltase, alkaline phosphatase, γ-glutamyl transferase, SOD and MDA contents were all determined using commercial kits.

### Enzyme linked immunosorbent assay (ELISA)

The ELISA assay was conducted according to the protocols described previously [35]. Briefly, the intestine and lung tissues were obtained and stored at − 80 °C until the assays were performed. Mouse sIgA and DAO ELISA kits were placed in room temperature for at least 20 min before use. The samples were added into each well followed by incubation for 2 h at 37 °C. Biotin-antibody was hereafter added after removing the liquid of each well, and incubated at 37 °C for 1 h. After washing with buffer, the horseradish peroxidase-conjugate was added into each well and incubated for additional 1 h at 37 °C. Subsequently, the chromogenic substrate was incubated for 15 min at 37 °C in the dark for color development. The absorbance was measured at the wavelength of 450 nm by a microplate reader (Thermo Fisher Scientific Inc., Waltham, MA, USA).

### 16S ribosomal RNA gene sequencing

The 16S ribosomal RNA (rRNA) gene sequencing was performed according to the protocols described previously [41]. Briefly, at the end of 28 days’ treatment, the fecal samples of animals were collected under sterile conditions and stored at − 80 °C before use. Total genomic DNA was extracted from samples and verified by 1% agarose gel electrophoresis. The bacterial 16S rRNA were amplified by the forward and reverse primers designed by adding a barcode to primer. The polymerase chain reaction (PCR) amplification reaction was performed on the ABI GeneAmp 9700 (Thermal cyclers from Applied Biosystems, CA, USA) with TransStart Fastpfu DNA Polymerase. The PCR products were excised from agarose and purified by AxyPrep DNA Gel Extraction Kit (Axygen Biosciences, CA, USA). Subsequently, the PCR products were quantified by QuantiFluor™-ST Blue Fluorescence Quantification System (Promega Co., WI, USA). The MiSeq library was constructed for preparation of the fragment DNA by TruSeq™ DNA Sample Prep Kit. At last, the raw sequence reads were obtained by Illumina MiSeq platform at Majorbio Bio Tech Co. Ltd (Shanghai, China).

### 16S ribosomal RNA gene sequencing analysis

The raw sequence reads were clustered into operational taxonomic units (OTUs) with 97% similarity on Majorbio’s cloud website at https://cloud.majorbio.com (Majorbio, Shanghai, China) based on the Usearch software programs (version 7.1). The alpha diversity indices, Shannon, Simpson, Chao, Ace and the observed species were calculated by Mothur software programs (version v.1.30.1). β-diversity was obtained by principal component analysis (PCA) and partial least squares discriminant analysis (PLS-DA) in the R software. The dominant phylotypes responsible for significant differences between two groups were assessed by linear discriminant analysis effect size (LEfSe). Linear discriminant analysis (LDA) score was set as 2.0. The enterotypes of microbiota were analyzed in the ade4, cluster and clustersim packages of R software. The relationship between sample and microbial community was visualized by the Circos-0.67-7 software. Phylogenetic tree on the Genus level was bootstrapped using Mega software (version 10.0). The network analysis on the OTU level and network correlation analysis were performed on the software of NetworkX. The PICRUSt was used to predict the functional composition of microbial community.

### Morris water maze

Morris water maze was used for the assessment of spatial learning and memory function [42]. Briefly, Morris water maze was performed after indicated treatment. The circular pool was divided into four equal quadrants and filled with water. The mice were put into the water maze for adaptation with a 60 s free swim without the platform before trials. The hidden platform test was conducted for 4 consecutive days. The swim path of each mouse was recorded by the video camera mounted above the pool. The swim distance, escapes latency and the swim speed were all obtained from the tracking system. Finally, the hidden platform was removed for post-training probe tests at the last day, the time spent in the target quadrant and the number of platform crossings were recorded during test.

### Open-field test

Open-field test was applied to assess the locomotor activity of animals [42]. In brief, the mouse was placed in the center of apparatus facing the same direction. The activity of each mouse in the apparatus was observed for 5 min. The apparatus was carefully cleaned with 75% ethanol between each trial. During the test, the total distance, distance moved in center and central square duration were all obtained from the tracking system with video camera mounted above the apparatus.

### Statistical analysis

All the experimental data were reported as mean ± standard error of the mean (S.E.M). Independent student-*t* test or non-parametric Mann–Whitney U test were applied to detect the statistically significant differences between two groups. The repeated-measure analysis of variance (ANOVA) was use to assess the statistical significance on the escape latency, swim distance and swim speed in the Morris water maze. All the statistical analysis was carried out using GraphPad Prism 8.0 (GraphPad Software, La Jolla, CA), and the statistical significance level was set at *p* value less than 0.05.

## Results

### Oral exposure to SiO_2_NPs led to spatial learning and memory impairment and locomotor inhibition

**Fig. 2:**
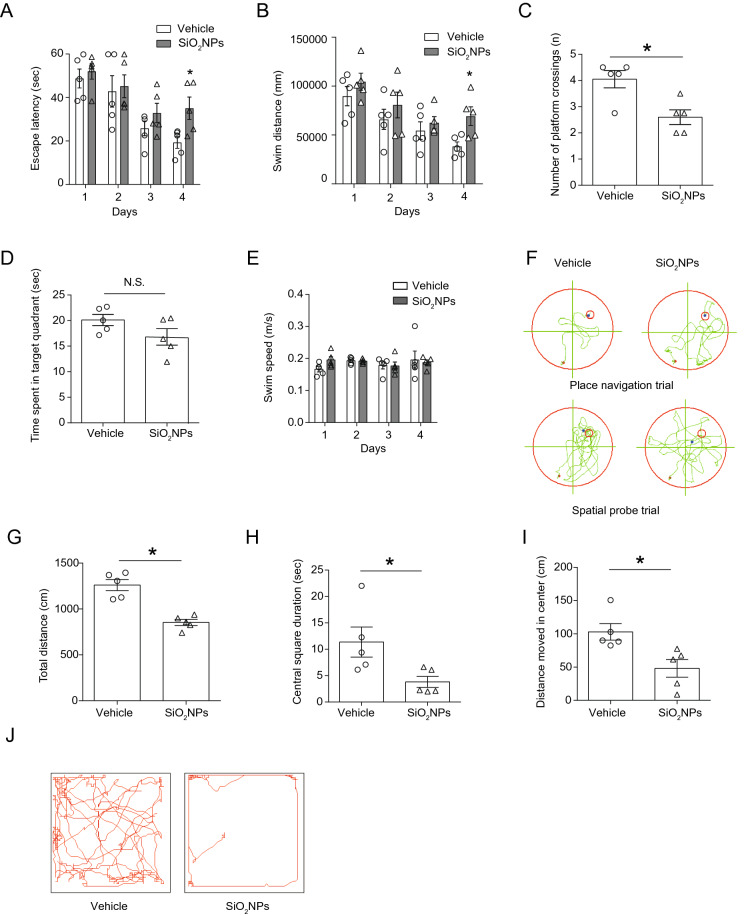
Oral exposure to SiO_2_NPs led to spatial learning and memory impairment and locomotor inhibition. After indicated treatment, Morris water maze and open filed test were used to evaluate the spatial learning and memory function and locomotor activity of mice, respectively. Effects of SiO_2_NPs on the escape latency were shown in (**A**). **B** Effects of SiO_2_NPs on the swim distance. **C** Effects of SiO_2_NPs on the number of platform crossings. **D** Effects of SiO_2_NPs on the time spent in target quadrant. **E** Effects of SiO_2_NPs on the swimming speed. **F** Representative track maps of vehicle mice and SiO_2_NPs-treated mice in the place navigation trial and spatial probe trial. **G** Effects of SiO_2_NPs on the total distance in the open field test. **H** Effects of SiO_2_NPs on the central square duration. **I** Effects of SiO_2_NPs on the distance moved in the center. **J** Representative track maps of two groups in the open field test. Data were shown as mean ± S.E.M. Statistical analysis was conducted by using repeated-measure ANOVA or independent student-*t* test or Mann–Whitney test. Asterisk * indicated *P* < 0.05

Morris water maze is a widely used and well-validated neurobehavioral test for the assessment of spatial learning and memory ability [43]. In this study, Morris water maze was carried out after treated with SiO_2_NPs for 28 days. As shown in Fig. 2A, the results demonstrated that the escape latencies of mice exposed to SiO_2_NPs were significantly higher than those of vehicle controls, indicating that the mice took more time to make first contact with the hidden platform. Similarly, the swim distances of SiO_2_NPs-treated animals were also much longer than those of vehicle controls (Fig. 2B). These data suggest that the spatial learning function of animal is remarkably impaired by SiO_2_NPs. In the probe test, the results revealed that the number of platform crossings was obviously reduced in SiO_2_NPs group as compared with vehicle group (Fig. 2C). The time spent in target quadrant was also slightly decreased in SiO_2_NPs-treated mice, but it did not reach the statistical significance (Fig. 2D). No significant changes were observed on the swim speed between two groups (Fig. 2E). Representative track maps of each group in Morris water maze were depicted in Fig. 2F. These obtained results together imply that oral exposure to SiO_2_NPs partially disrupt the spatial learning and memory function of mice.

Open field test is a classical test used for evaluation of locomotor activity in animal models [44]. To further evaluate the effect of SiO_2_NPs on the locomotor activity, the open field test was conducted. The results illustrated that the total distance in the open-field apparatus was sharply declined in SiO_2_NPs-treated mice as compared with vehicle controls (Fig. 2G). Meanwhile, the central square duration and distance moved in center were both notably declined in SiO_2_NPs-treated group in comparison to control group (Fig. 2H, I). Representative track maps of each group in open field test were depicted in Fig. 2J. These results together specify that oral exposure to SiO_2_NPs significantly inhibits the locomotor activity of animals.

### Oral exposure to SiO_2_NPs caused the disturbance of gut microbiota and their associated biological functions

**Fig. 3:**
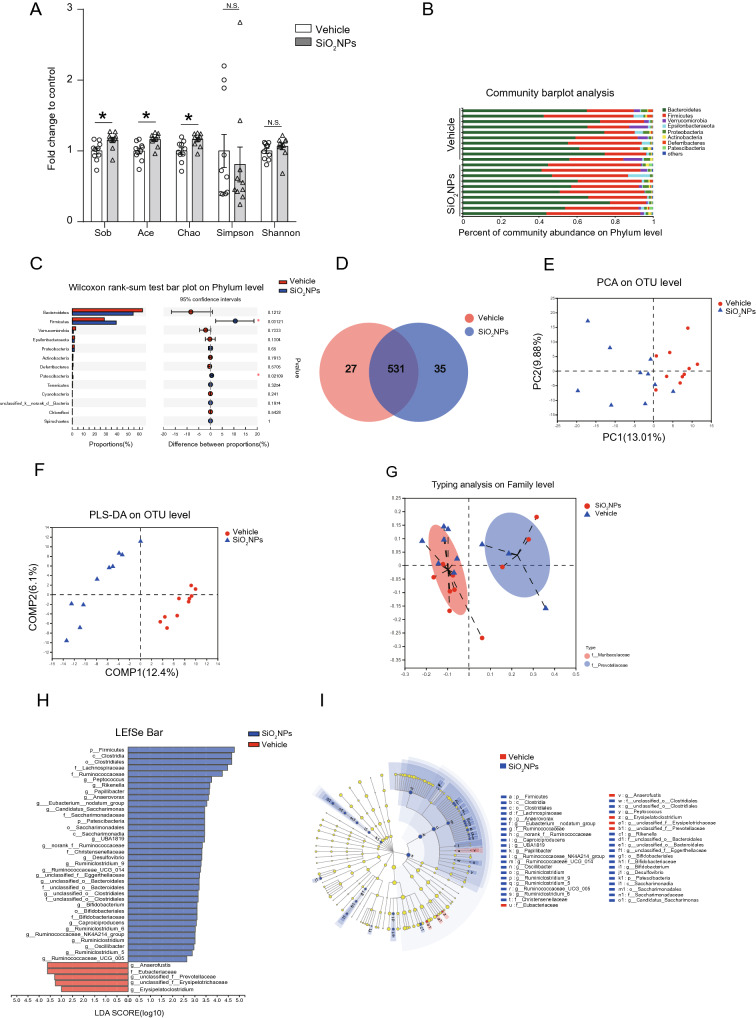
Oral exposure to SiO_2_NPs caused the disturbance of gut microbiota and their associated biological functions. The fecal samples of two groups were collected and subjected to 16S ribosomal RNA (rRNA) gene sequencing. **A** Sobs, Ace, Chao, Simpson, and Shannon were determined to assess the α-diversity of gut microbiota. Data were reported as mean ± S.E.M. Statistical analysis was calculated by using independent student-*t* test. Asterisk * indicated *P* < 0.05. **B** The percent of community abundance on phylum level was detected in two groups. The differences on the community abundance on phylum level were analyzed and shown in (**C**). **D** Venn diagram showed the overlap of core microbiota between vehicle group and SiO_2_NPs-treated group. **E** The dissimilarities in microbial composition on OTU level was determined by principal component analysis (PCA). **F** Partial least squares discrimination analysis (PLS-DA) was used to detect the gut microbial community compositions between two groups. Bacterial clades and biologically consistent difference (LDA score > 2.0) were assessed by Linear discriminant analysis (LDA) coupled with effect size (LEfSe) (**H**, **I**). Typing analysis on family level was shown in (**G**)

After exposure to SiO_2_NPs, the bacterial DNA in feces were extracted and analyzed by 16S rRNA gene sequencing. The sequences were clustered into OTU based on a 97% similarity threshold. Obtained total number of OTU was 593. The coverage indices of vehicle group and SiO_2_NP-treated group were 0.9982 ± 0.0003 and 0.9979 ± 0.0002. The rarefaction curves of two groups both showed clear asymptotes (Additional file 1: Figure S1A). These results together suggest a near-complete sampling of the community. α-diversity is an intuitive index for evaluation of microbial diversity. In this study, the results showed that the Sobs, Ace, and Chao were all significantly enhanced in SiO_2_NPs-treated group, but no significant changes were observed on two other indices, Simpson, and Shannon (Fig. 3A). These data together imply that exposure to SiO_2_NPs can partially affect α-diversity of gut microbiota.

The percent of community abundance on phylum level in two groups was presented in Fig. 3B. In the vehicle group, the dominant bacteria were *Bacteroidetes* (62.68%), *Firmicutes* (28.58%), *Verrucomicrobia* (3.11%), *Epsilonbacteraeota* (2.09%), *Proteobacteria* (1.79%), whereas the SiO_2_NPs group displayed a high relative abundance of *Bacteroidetes* (54.30%), followed by *Firmicutes* (39.17%), *Epsilonbacteraeota* (1.82%), *Proteobacteria* (1.80%) and *Verrucomicrobia* (1.03%). The abundances of *Firmicutes* and *Patescibacteria* in the SiO_2_NPs-treated group were significantly higher than those in the vehicle control group (Fig. 3C). As shown by the Venn’s diagram in Fig. 3D, two groups shared the compositional overlap of 531 core microbiota. These overlapping phylotypes contributed to 95.16% (531/558) and 93.81% (531/566) of vehicle group and SiO_2_NPs-treated group, respectively. Principal component analysis (PCA) was employed to detect the dissimilarities in microbial composition on OTU level. As shown in Fig. 3E, SiO_2_NPs-treated samples were primarily concentrated on the left side, whereas the samples in the vehicle control group presented mainly on the right side. Partial least squares discrimination analysis (PLS-DA) further clearly distinguished SiO_2_NPs-treated samples from vehicle samples, indicating that there were significant differences on the gut microbial community compositions between two groups (Fig. 3F). Two enterotypes were observed in this study on the family level, one was *f_Muribaculaceae*, the other one was *f_Prevotellaceae*. No significant difference was shown on the enterotype between two groups (Fig. 3G). Linear discriminant analysis (LDA) coupled with effect size (LEfSe) established 41 bacterial clades showing statistically significant and biologically consistent differences (LDA score > 2.0) from phylum to genus level (Fig. 3H, I).

**Fig. 4:**
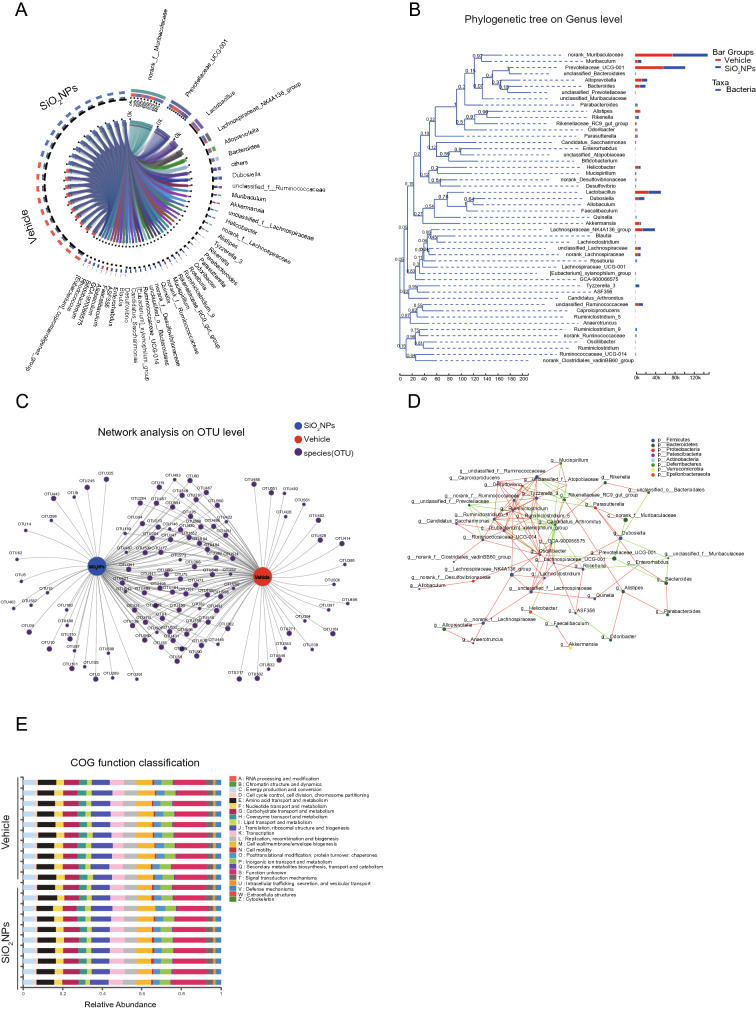
Oral exposure to SiO_2_NPs caused the disturbance of gut microbiota and their associated biological functions. **A** The correlations between microbiota and samples in the vehicle group and SiO_2_NPs group was detected using Circos analysis. **B** The phylogenetic tree was used to show the evolutionary relationships among bacteria on the genus level. Network analysis at the OTU level was shown in (**C**). The relationship of microbial communities each other was displayed in (**D**). **E** The clusters of orthologous groups (COGs) analysis displayed the top increased and the top decreased functional abundances of microbial community

Circos analysis was carried out to visualize the correlations between microbiota and samples in vehicle group and SiO_2_NPs group (Fig. 4A). The evolutionary relationships among bacteria on the genus level were displayed in the phylogenetic tree (Fig. 4B). Network analysis was performed at the OTU level and shown in Fig. 4C. The relationships among microbial communities were displayed in Fig. 4D. The red line indicated the negative correlation and the green line presented positive correlation between each species at genus level. The size of dot indicated the degree of relevance with other species. The PICRUSt was further used to predict the functional composition of a microbial community’s metagenome. In the analysis on the clusters of orthologous groups (COGs), the results revealed that the top three increased functional abundances of microbial community were cytoskeleton, cell motility and signal transduction mechanisms, and the top three decreased functional abundances of microbial community were extracellular structures, RNA processing and modification and chromatin structure and dynamics (Fig. 4E). In the Kyoto Encyclopedia of Genes and Genomes (KEGG) function analysis, the top three elevated functional abundances were KO03406 (methyl-accepting chemotaxis protein), KO02529 (LacI family transcriptional regulator), KO02003 (putative ABC transport system ATP-binding protein), whereas the top three reduced functional abundances were KO6142 (outer membrane protein), KO2014 (iron complex outer membrane receptor protein), KO3773 (FKBP-type peptidyl-prolyl *cis*–*trans* isomerase FklB) in the top 50 highest abundances of KEGG orthology (KO) (Additional file 1: Fig. S1B). On the KEGG pathway level 2, carbohydrate metabolism, global and overview maps and amino acid metabolism were the top increased functional abundances, while circulatory system and substance dependence were the most decreased functional abundances (Additional file 1: Fig. S1C). Taken together, these results suggest oral exposure to SiO_2_NPs may lead to the disturbance of gut microbiota and their associated biological functions.

### Oral exposure to SiO_2_NPs did not result in inflammation in the intestine but remarkably damage the tissue integrity

**Fig. 5:**
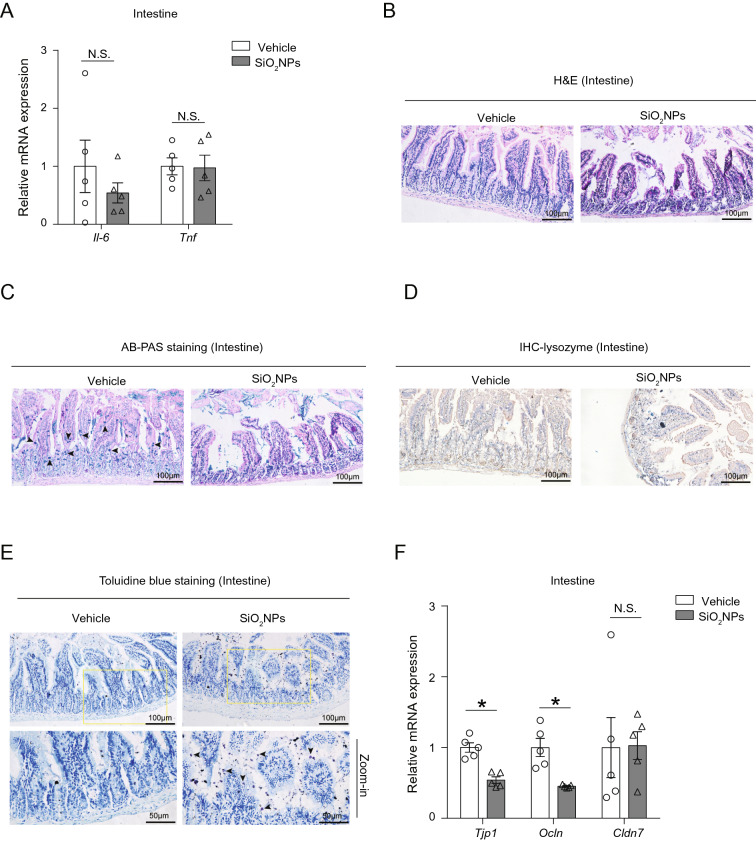
Oral exposure to SiO_2_NPs did not result in inflammation in the intestine but remarkably damage the tissue integrity. **A** Effects of SiO_2_NPs on the *Il-6* and *Tnf* mRNA expressions in gut were shown. **B** The morphological alterations of gut tissues were determined by H&E staining after oral exposure to SiO_2_NPs. **C** AB-PAS staining assay was used to evaluate the number and size of goblet cells between vehicle control and SiO_2_NPs-treated animals. **D** Effects of SiO_2_NPs on the Paneth cell compartment and the overall granule content. **E** The number of toluidine blue-positive mast cells were evaluated by Toluidine blue staining. **F** Effects of SiO_2_NPs on the intestinal tight junction was measured by the mRNA expressions of *Tjp1*, *Ocln* and *Cldn 7*. Scale bar, 100 μm or 50 μm. Data were reported as mean ± S.E.M. Statistical analysis was performed by using independent student-*t* test. Asterisk * indicated *P* < 0.05

Recently, studies have suggested a new role for gut microbiota in the regulation of intestinal inflammation [45, 46]. To investigate if SiO_2_NPs-induced dysbiosis of gut microbiota causes to the inflammation in the intestinal tract, the mRNA expressions of *Il-6* and *Tnf* were determined. As shown in Fig. 5A, unapparent alterations on these two genes after treating of mice with SiO_2_NPs were spotted. Next, the tissue integrity of intestine was assessed by histopathological examination. The obtained results in H&E staining assay illustrated that, exposure to SiO_2_NPs caused the abnormal villous shortening, and the tips of villi appeared ragged, irregularly shaped, or completely destroyed (Fig. 5B). In the AB-PAS staining assay, we found that the number and size of goblet cells were both reduced significantly in SiO_2_NPs-treated animals (Fig. 5C). Paneth cells were further identified using a specific anti-lysozyme antibody. Similar trend was observed in Paneth cells after exposure to SiO_2_NPs. Our results demonstrated that the size of the Paneth cell compartment and the overall granule content were obviously reduced in the mice treated with SiO_2_NPs (Fig. 5D). On the contrary, the number of toluidine blue-positive mast cells were increased significantly in response to SiO_2_NPs exposure (Fig. 5E). To further investigate the effects of SiO_2_NPs on the intestinal tight junction, the mRNA expressions of *Tjp1*, *Ocln*, *Cldn 7* were measured. As shown in Fig. 5F, the results demonstrated that the mRNA expressions of *Tjp1* and *Ocln* were both sharply reduced in SiO_2_NPs-treated group as compared with vehicle group. But no significant alteration was observed on the mRNA expression of *Cldn 7*. These results together indicate that oral exposure to SiO_2_NPs for 28 days can damage the integrity of tissue and cause inconspicuous inflammation response in the intestine.

### Oral exposure to SiO_2_NPs partially affected the activities of intestinal digestive enzymes and immune functions

**Fig. 6:**
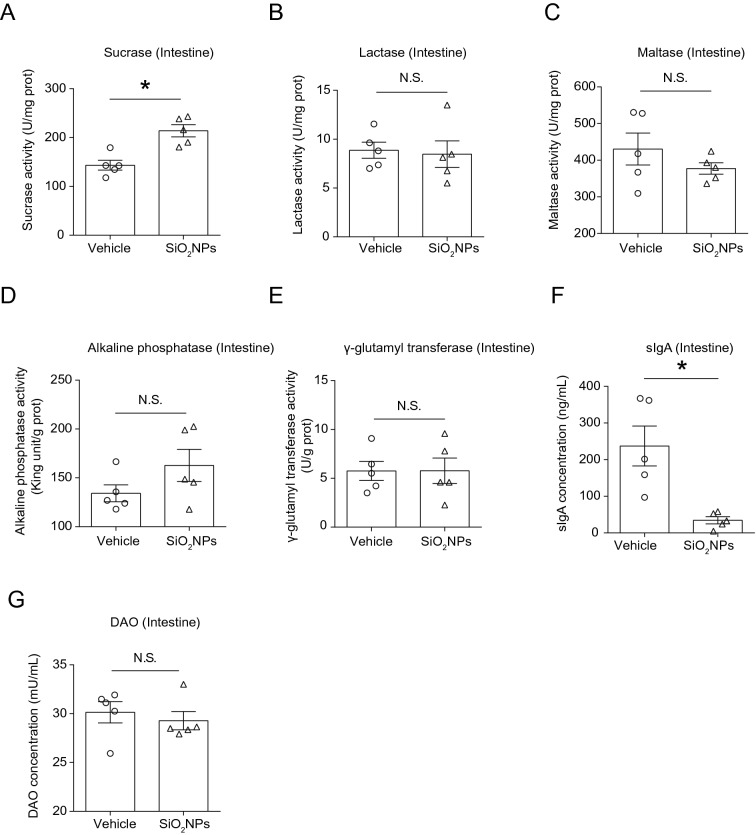
Oral exposure to SiO_2_NPs partially affected the activities of intestinal digestive enzymes and immune functions. After indicate treatment, the effects of SiO_2_NPs on the activities of typical digestive enzymes, including sucrase, lactase, maltase, alkaline phosphatase and γ-glutamyl transferase were shown in (**A**–**E**). **F** Effects of SiO_2_NPs on the levels of sIgA in the gut tissues were detected using ELISA assay. **G** Effects of SiO_2_NPs on the DAO contents were determined by ELISA. Data were reported as mean ± S.E.M. Statistical analysis was performed by using independent student-*t* test. Asterisk * indicated *P* < 0.05

Digestive enzymatic activity plays a critical role in maintaining the normal microbial ecology of the gastrointestinal system [47]. Therefore, the activities of typical digestive enzymes, including sucrase, lactase, maltase, alkaline phosphatase and γ-glutamyl transferase, were evaluated after SiO_2_NPs administration. The results revealed that the activity of sucrase was dramatically elevated in SiO_2_NPs-treated group when compared with vehicle control group (Fig. 6A). However, no significant changes were observed in other kind of digestive enzymes’ activities (Fig. 6B–E). The obtained results imply that oral exposure to SiO_2_NPs may partially affect the activity of intestinal digestive enzyme, but this influence is very limited.

The gastrointestinal tract is a highly complex system that has distinct functions not only in digestion, but also in immune homeostasis [10, 48]. sIgA is an essential part of intestinal barrier. It can bind to antigens and increase the capture of antigens, thereby strengthening the immune function of intestinal barrier [49]. In response to exogenous damage, the gastrointestinal tract commonly generates sIgA immune response [10]. In this study, the results showed that treatment of SiO_2_NPs, the levels of sIgA were sharply declined as compared with vehicle controls (Fig. 6F). Since DAO is closely associated with microbial induction of intestinal sIgA [49, 50], the content of DAO was further detected using ELISA. Nevertheless, we did not find any significant alteration in the DAO content between two groups (Fig. 6G). Collectively, these results identify sIgA as previously unrecognized immune mediators of microbe–host interplay in the intestine after SiO_2_NPs administration.

### Oral exposure to SiO_2_NPs did not result in brain inflammation but might cause the brain damage via gut–brain axis

**Fig. 7:**
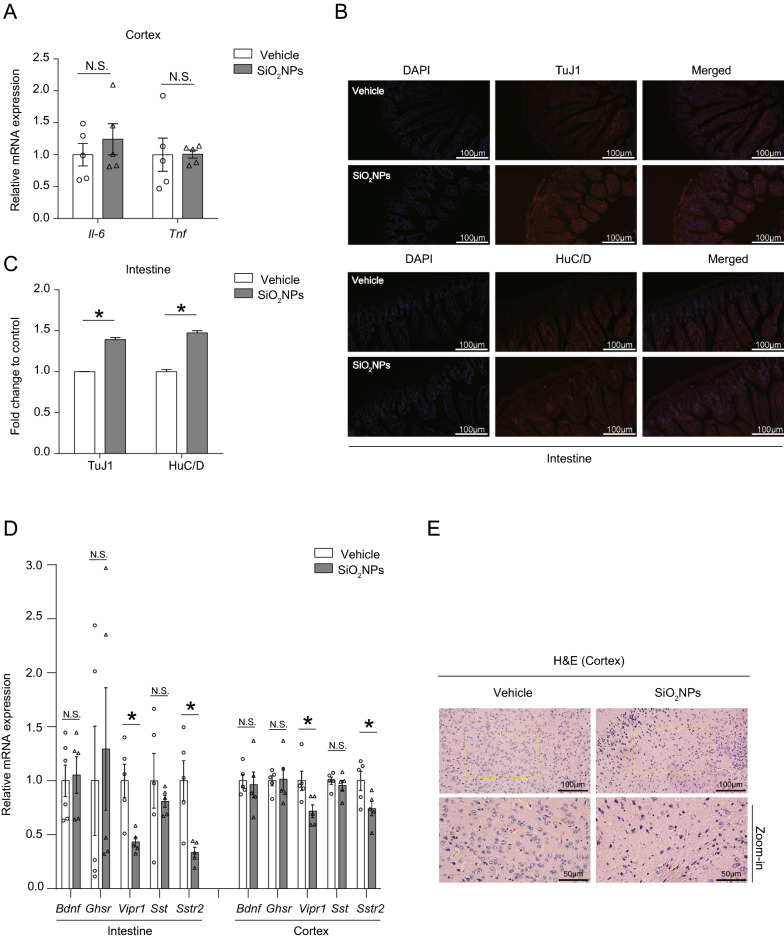
Oral exposure to SiO_2_NPs did not result in brain inflammation but might cause the brain damage via gut–brain axis. **A** Effects of SiO_2_NPs on the mRNA expressions of inflammation indicators, *Il-6* and *Tnf*, in the cerebral cortex tissues. The expressions of HuC/D and TuJ1, both of which were specific enteric neuron markers, were detected by immunofluorescence assay. The representative images and fluorescence intensity of HuC/D and TuJ1 expression were displayed in (**B** and **C**), respectively. Scale bar, 100 μm. **D** Effects of SiO_2_NPs on the mRNA expressions of *Vipr1*, *Sstr2*, *Bdnf*, *Sst* and *Ghsr* in the cortex and gut. **E** The pathological changes of SiO_2_NPs-treated animals were measured using H&E staining. Scale bar, 100 μm and 50 μm. Data were reported as mean ± S.E.M. Statistical analysis was performed using independent student-*t* test. Asterisk * indicated *P* < 0.05

To investigate whether exposure of SiO_2_NPs triggers neurobehavioral impairments in animals via gut–brain axis, the levels of its related indicators were assessed. At first, the mRNA expressions of inflammation indicators, *Il-6* and *Tnf*, were both detected in the cerebral cortex, where motor and memory functions were initially processed. As shown in Fig. 7A, the results elaborated that either *Il-6* or *Tnf* mRNA expression did not change significantly after exposure of SiO_2_NPs, indicating that oral administration of SiO_2_NPs might not result in the neuroinflammation in the cerebral cortex. Intriguingly, our results revealed that oral exposure to SiO_2_NPs remarkably increased the expressions of HuC/D and TuJ1 in the gut, indicating the excitement of enteric neurons induced by SiO_2_NPs (Fig. 7B, C). Gut–brain peptides are the key signaling molecules that are involved in the regulation of gut–brain axis. Alterations of gut-derived peptides are highly related with the neurobehavioral dysfunction. The results demonstrated that the mRNA expression of *Vipr1* was obviously reduced in both gut and cortex tissues of SiO_2_NPs-treated group as compared with control group (Fig. 7D). Similar trends were observed on the *Sst2* expressions in the gut and cortex tissues, showing that the mRNA expressions of *Sstr2* was remarkably reduced in mice exposed to SiO_2_NPs (Fig. 7D). However, the levels of *Bdnf*, *Sst* and *Ghsr* expressions did not change significantly (Fig. 7D). The pathological changes were observed under light microscope in SiO_2_NPs-treated animals, manifested by reduced neuronal cells, cell shrinkage, rupture and deformation, chromatin condensation and nuclear fragmentation (Fig. 7E). These results together suggest that oral administration of SiO_2_NPs is capable of inducing brain damage via gut–brain axis by specific chemical substances originated from gut, but does not trigger neuroinflammation in the cerebral cortex of mice.

### Oral exposure to SiO_2_NPs did not affect the gut–lung axis and gut-liver axis

**Fig. 8:**
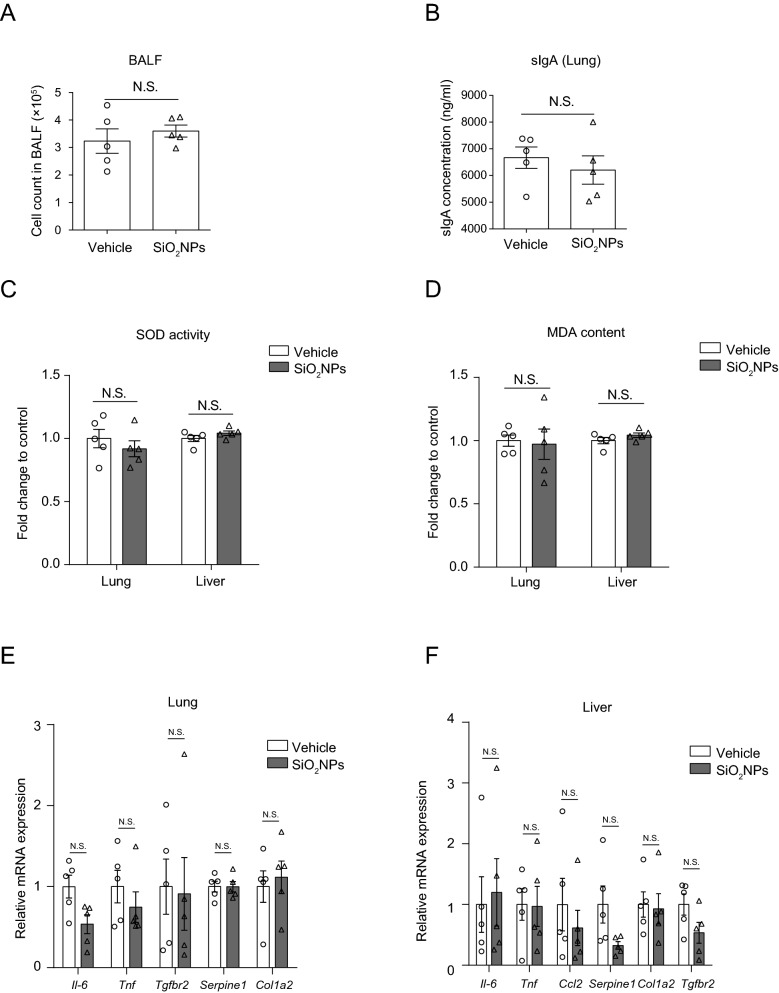
Oral exposure to SiO_2_NPs did not affect the gut-lung axis and gut liver axis. **A** Effects of SiO_2_NPs on the cell count in bronchoalveolar lavage fluid. **B** The levels of sIgA in the lung tissues of two groups were measured by ELISA assay. Effects of SiO_2_NPs on the activities of SOD and contents of MDA in the lung and liver tissues were displayed in (**C **and** D**). Effects of SiO_2_NPs on the mRNA expressions of *Il-6, Tnf Col1a2*, *Tgfbr2*, *Ccl2* and *Serpine1* in the lung and liver tissues were shown in (**E** and **F**). Data were reported as mean ± S.E.M. Statistical analysis was conducted by using independent student-*t* test. Asterisk * indicated *P* < 0.05

To determine whether oral exposure to SiO_2_NPs distinctively disrupt the gut–brain axis and therefore resulting in the neurobehavioral impairments such as spatial learning and memory and locomotor inhibition, the gut–lung axis and gut–liver axis related indicators were all evaluated. As shown in Fig. 8A, the cell count number in bronchoalveolar lavage fluid did not alter after SiO_2_NPs administration. Meanwhile, we did not find notably alteration on the level of sIgA in the lung tissue collected from SiO_2_NPs-treated mice (Fig. 8B). Both the activities of SOD and contents of MDA in the lung and liver tissues did not change significantly in SiO_2_NPs-exposed animals in comparison to vehicle controls (Fig. 8C, D). The mRNA expressions of *Il-6* and *Tnf* in the two groups did not show any significant difference. There was no significant alteration on the mRNA expression of *Ccl2* in the liver between two groups. No significant changes were observed on the mRNA expressions of *Col1a2*, *Tgfbr2* and *Serpine1*in the lung and liver tissues, all of which were the indicators involved in the regulation of gut–lung axis or gut–liver axis (Fig. 8E, F). Collectively, these findings indicate that exposure of SiO_2_NPs does not obviously affect the gut–lung axis and gut–liver axis.

## Discussion

Oral route of exposure to SiO_2_NPs is a vital consideration due to their deliberate addition to food and unintentional ingestion from contaminated environment [3, 11]. In the past decade, awareness has been raised on the behavior and interaction of SiO_2_NPs in the gastrointestinal tract [51]. However, in absence of robust evidence on the gastrointestinal uptake and distribution of SiO_2_NPs, it is now challenging to evaluate the potential gut health risk of foodborne SiO_2_NPs accurately. Therefore, in the present study, the young animals were orally treated with SiO_2_NPs, and the potential effects of SiO_2_NPs on the gastrointestinal environment and its associated functions were assessed. No significant changes were observed on the body weight between two groups (data not shown). Intriguingly, our results illustrated that, in addition to evoking dysfunctions and injuries in the gut, ingested SiO_2_NPs exhibited a moderate to severe impact on the gut microbial communities, and thereby resulting in spatial learning and memory impairments and locomotor inhibition via distinctive microbiota–gut–brain axis. Considering the long-term exposure of SiO_2_NPs via food, the potential effects of SiO_2_NPs on the gut microbiota and its related brain functions should be seriously concerned in health risk assessment, especially for the infants or children who are susceptible to the neurotoxicity of nano-sized particles.

Gut microbiota plays important roles in regulation of gastrointestinal functions [29, 45, 46, 48]. For instance, the microbes within the gut are essential to facilitate the digestion and produce vitamins, which provide a good foundation for human health. The gut microbiome is also indispensable for the development of intestinal epithelium as well as the host immune defense against pathogens. All of these biological functions of gut microbiota contribute greatly to lifelong maintenance of gastrointestinal microenvironment homeostasis [29, 48]. Conversely, a shift in the gut microbiota is related to the onset and/or progression of various diseases [30, 31, 45, 46]. Herein, our results demonstrated that exposure to SiO_2_NPs not only resulted in a significant shift in the composition of microbial communities, but also drastically enhanced the microbial species and diversity within the gut. Similar phenomenon was observed in the CD-1 mice orally administrated with SiO_2_NPs for 7 days [52]. Such an unexpected effect may due to the low absorption rate of gastrointestinal tract for precipitated or fumed silicate [10]. Another explanation is that the interactions between nanomaterials and microbiome may depend on the sampling area. The microbial composition can be very different in different regions of gastrointestinal tract [10, 51], and there may be a bias to determine the microbial composition in the intestine.

Beyond the gut, it is well established that the gut microbiota can modulate the function of other organs, such as brain, liver, and lung etc. [29, 53, 54]. The bidirectional communication that is enabled by many bacterial metabolites, gut-derived chemicals, peptides. These gut-originated substances can significantly affect distant organs either directly through systemic circulation or indirectly by signaling via vagus nerve or chemicals from the gut [29, 46, 48, 53, 54]. In this study, the obtained results revealed that, the enteric neurons were excited after SiO_2_NPs administration, and the mRNA expressions of *Vipr1* and *Sstr2* were significantly increased by SiO_2_NPs, accompanied with pathological changes and neurobehavioral impairments. These data may together support the hypothesis that exposure to SiO_2_NPs by oral treatment causes the spatial learning and memory deficits and locomotor inhibition by disrupting the gut–brain axis. On one hand, the disrupted microbiota may influence the physiological processes of specific gut-originated substances through their effects on the central nervous system. On the other, the damaged brain also plays a critical part in reshaping the microbiota that may be harmful for its metabolic activities. Intriguingly, our findings illustrated that treatment of SiO_2_NPs did not show any impacts on the gut–liver and gut–lung axis, manifested by the unchanged indicators determined in either lung or liver tissues in both two groups. These findings will represent the basis for better understanding how exposure to SiO_2_NPs distinctively shape the interactions between microbiome and neuronal functions.

Notably, previous reports have proved the capacity of SiO_2_NPs to translocate into the brain after intranasal instillation or intravenous injection [19, 26]. They can gradually accumulate in the different regions of brain due to the limited excretion of SiO_2_NPs in the body, and thereby resulting in injuries to neuronal cells. Based on this observation, recent investigations have focused on the role of SiO_2_NPs on brain functions. Either injected intravenously, or intranasal instillation of SiO_2_NPs, leads to the accumulation of nanoparticles in the brain, and ultimately causing the neurodegeneration-like changes in behaviors, such as spatial learning and memory impairment [19, 26]. Other previous studies have demonstrated that the neurotoxic effects caused by administering of SiO_2_NPs, specifically occurred by the elevation in oxidative stress and activation of microglial functions, leading to highly negative impacts on the neurons [24, 25, 28, 55]. However, to date, whether SiO_2_NPs is capable of translocating in the brain after oral administration has not been established, and the detailed mechanisms underlying how SiO_2_NPs exposure induces cognitive dysfunctions remain substantially unclear. Herein, the potential limitation is that we can not exclude the possibility that accumulated SiO_2_NPs in the brain directly triggers the neuronal damage. Notwithstanding, the current findings presented in this study will still provide valuable information in the understanding of gut–brain axis involved in SiO_2_NPs-induced neurobehavioral impairments.

In this study, oral exposure to SiO_2_NPs for 28 days in young mice was shown to damage the intestinal and cerebral cortex tissues. Similar trends were reported in the previous investigations conducted by You et al. [26] and Salem et al. [56], in both of which neuropathological changes were presented in SiO_2_NPs-exposed animals. Interestingly, we did not find that oral exposure of SiO_2_NPs directly triggered inflammatory response in these two regions. Conversely, increased levels of neuroinflammation in the cerebral cortex and hippocampus, manifested by the enhancement of *Il-6* and *Il-1β* mRNA expressions, were observed in mice intranasal administrated with SiO_2_NPs [26]. Such inconsistency on the induction of inflammation may due to the different routes of exposure as well as the dosage used in the animal model. Furthermore, the results also clearly elucidated that the number of toluidine blue-positive mast cells was elevated markedly after SiO_2_NPs exposure. Because of the reciprocal interactions between mast cells and neuroendocrine immune network, the decreased number of mast cells may partially contribute to the alterations of gut–brain signaling chemicals, such as *Vipr1* and *Sstr2*. In addition, mast cells are also reported to be involved in the modulation of digestive secretions by release of histamine via gut–brain axis [57]. In this exposure model of SiO_2_NPs, our results precisely demonstrated that the activities of sucrase were increased significantly. Given that the activity of sucrase is usually elevated as the concentration of sucrose increases in the intestine, the enhancement of its activity indicate that SiO_2_NPs may adaptively increase the level of sucrose, which is of capacity to lower gut microbial diversity. Another cause for the improvement of digestive enzyme activity perhaps is that SiO_2_NPs modified the secretion of bacterial enzyme.

Intestinal mucosal barrier is the first line of physical defense against external substances. This barrier is a heterogeneous entity mainly composed of mechanical, biochemical, and immune barriers [58]. The obtained results explicated that the mRNA expressions of *Tjp1* and *Ocln*, both of which were tight junction proteins, were sharply reduced by SiO_2_NPs. In H&E staining assay, pathological changes were also observed in SiO_2_NPs-treated animals. These findings together indicate the mechanical barrier of gut is significantly damaged by SiO_2_NPs, which may further lead to the disturbance of gut microenvironment and increase the sensitivity of gut to exogenous stimuli. Meanwhile, the sharply reduced contents of sIgA, Paneth cell compartment and overall granule also signify that the capacity of immune host defenses in the gut is weaken after oral exposure of SiO_2_NPs. Notably, the disrupted barrier function may change the intestinal permeability that harmful substances or pathogens are easily to pass through epithelial barrier, thereby causing the adverse effects in different distant organs.

The limitations of this study should be considered. At first, in animal studies, the dosage and duration of oral administration of SiO_2_NPs are important factors for mimicking exposure conditions to which humans may be exposed through the daily dietary intake [10]. Thus, we herein calculated the dosage of SiO_2_NPs according to the upper limit of SiO_2_ added in the food additive. Although this dosage seems unusually high, it is needed for health risk assessment of foodborne SiO_2_NPs. Secondly, due to the lack of cogent evidence obtained from germ-free animals treated with SiO_2_NPs, the causal effects of gut microbiota on the brains and behaviors should be verified in the further studies.

## Conclusion

To the best of our knowledge, this is the first work that elucidates that oral exposure to SiO_2_NPs results in the spatial learning and memory impairments and locomotor inhibition via distinctive gut–brain axis. The implications of this study include the novel idea that proper maintenance and coordination of gut functions may be critical for protection against the neurotoxicity of foodborne SiO_2_NPs because of the multifaceted system of gut–brain bidirectional communication. This study may also provide valuable scientific evidence for policy makers to evaluate the safety of SiO_2_NPs in infant food applications.

## Supplementary Information

**Additional file 1: Figure S1.** (A) The rarefaction curves of two groups were displayed. (B) The Kyoto Encyclopedia of Genes and Genomes (KEGG) function analysis obtained top 3 elevated functional abundances and top 3 reduced functional abundances in top 50 highest abundances of KEGG orthology (KO). (C) Functional abundances were measured on the KEGG pathway level 2.

**Additional file 2: Table S1.** Primer sequences of target genes.

## Data Availability

The datasets used and/or analyzed during the current study are available from the corresponding author on reasonable request.
