# Supplementary figures and images for: Silicon dioxide nanoparticles induced neurobehavioral impairments by disrupting microbiota–gut–brain axis
Source: J Nanobiotechnology. 2021 Jun 10;19:174. doi: 10.1186/s12951-021-00916-2 (PMC8194163; doi:10.1186/s12951-021-00916-2)

Figure S1

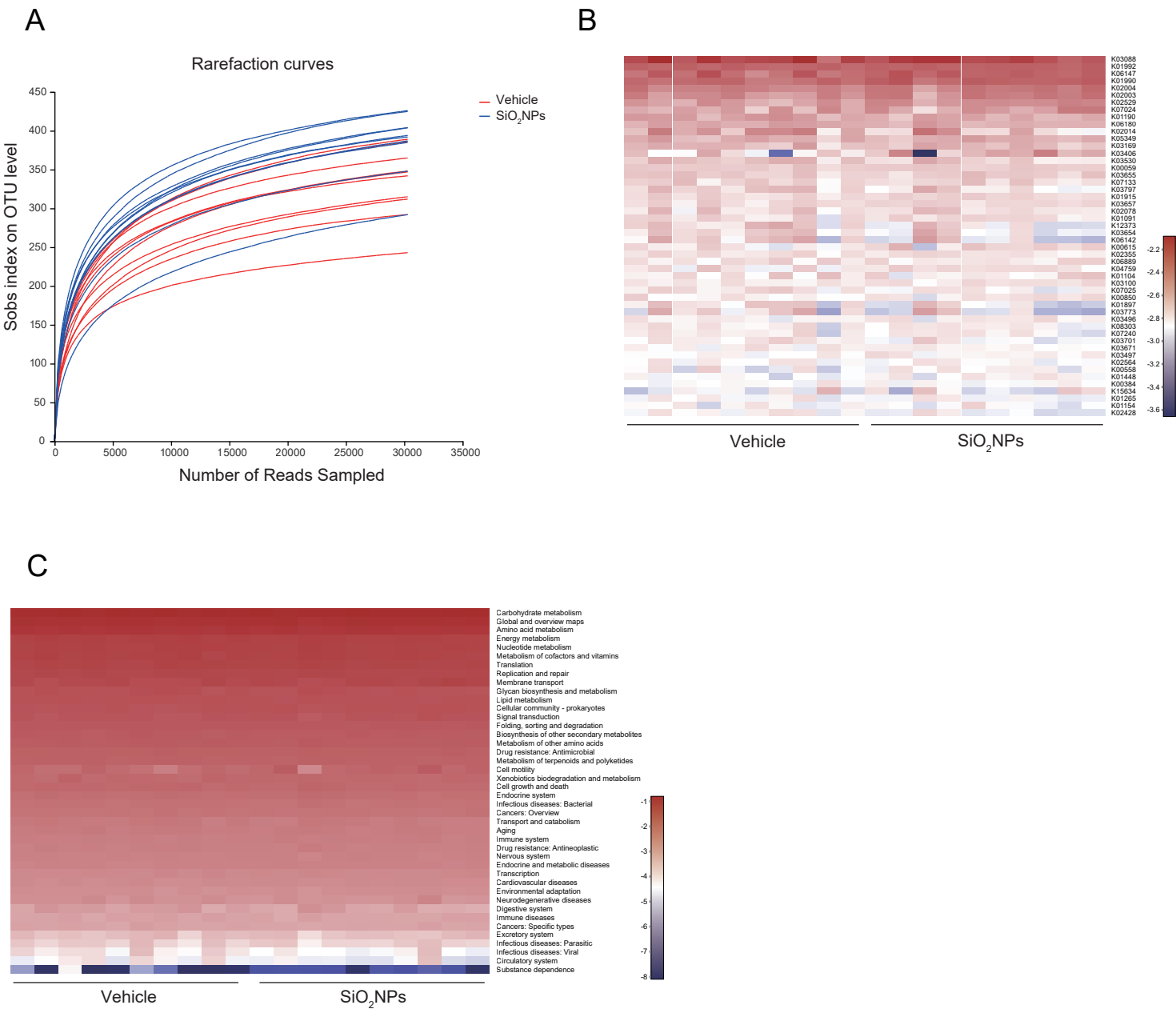

Supplement: Supplementary file 1 — Additional file 1: Figure S1. (A) The rarefaction curves of two groups were displayed. (B) The Kyoto Encyclopedia of Genes and Genomes (KEGG) function analysis obtained top 3 elevated functional abundances and top 3 reduced functional abundances in top 50 highest abundances of KEGG orthology (KO). (C) Functional abundances were measured on the KEGG pathway level 2. [file 12951_2021_916_MOESM1_ESM.pdf]
