# Supplementary material for: Silicon dioxide nanoparticles induced neurobehavioral impairments by disrupting microbiota–gut–brain axis
Source: J Nanobiotechnology. 2021 Jun 10;19:174. doi: 10.1186/s12951-021-00916-2 (PMC8194163; doi:10.1186/s12951-021-00916-2)
Supplement: Supplementary file 2 — Additional file 2: Table S1. Primer sequences of target genes. [file 12951_2021_916_MOESM2_ESM.doc]

Table S1. Primer sequences of target genes

| Gene | Forward (5’-3’) | Reverse (5’-3’) |
| --- | --- | --- |
| *Il-6* | CATCCAGTTGCCTTCTTG | ATTAAGCCTCCGACTTGT |
| *Tnf* | TCTCAGCCTCTTCTCATTC | GCCATTTGGGAACTTCTC |
| *Ocln* | TGGCTATGGAGGCGGCTATGG | AAGGAAGCGATGAAGCAGAAGGC |
| *Cldn7* | GCTTCTTAGCCATGTTTG | GTGACAATCTGATGACCA |
| *Tjp1* | AGCTGCCTCGAACCTCTACTCTAC | GCCTGGTGGTGGAACTTGCTC |
| *Bdnf* | CTCCTCTACTCTTTCTGC | CACTCGCTAATACTGTCA |
| *Col1a2* | CTCAGAACATCACCTACC | TCGACTAGGACAGAGTAG |
| *Tgfbr2* | GCTCTAACATCCTAGTGAA | CATCCACAGACAGAGTAG |
| *Ccl2* | ATGAGATCAGAACCTACAACT | ATGAGATCAGAACCTACAACT |
| *Vipr1* | CTATAACATCAGCCGTAACT | GCATCGTAGAACTCAGTC |
| *Ghsr* | AGAGAAAGGAATCCAAGAAG | CATGCTGCTGATACTGAG |
| *Sst* | CCAACCAGACAGAGAATG | ACAGGATGTGAATGTCTTC |
| *Serpine1* | GACTCCTTTCTTAGAGGC | GTGGTGAACTCAGTGTAG |
| *Sstr2* | CAACATCTACATCCTTAACCT | GATACTGGTGAACTGATTGA |
| *β-actin* | CGTTGACATCCGTAAAGA | CAGAGCAGTAATCTCCTTC |
